# Early Measurement of ROX Index in Intermediary Care Unit Is Associated with Mortality in Intubated COVID-19 Patients: A Retrospective Study

**DOI:** 10.3390/jcm11020365

**Published:** 2022-01-12

**Authors:** Alexandre Leszek, Hannah Wozniak, Amélie Giudicelli-Bailly, Noémie Suh, Filippo Boroli, Jérôme Pugin, Olivier Grosgurin, Christophe Marti, Christophe Le Terrier, Hervé Quintard

**Affiliations:** 1 Department of Medicine, Division of General Internal Medicine, Geneva University Hospitals, 1205 Geneva, Switzerland; alexandre.leszek@hcuge.ch (A.L.); olivier.grosgurin@hcuge.ch (O.G.); christophe.marti@hcuge.ch (C.M.); 2 Department of Acute Medicine, Intensive Care Unit, Geneva University Hospitals, 1205 Geneva, Switzerland; hannah.wozniak@hcuge.ch (H.W.); amelie.bailly@hcuge.ch (A.G.-B.); noemie.suh@hcuge.ch (N.S.); filippo.boroli@hcuge.ch (F.B.); jerome.pugin@hcuge.ch (J.P.); christophe.leterrier@hcuge.ch (C.L.T.)

**Keywords:** ROX index, COVID-19 mortality predictive factor, COVID-19 intermediate care unit patients, acute hypoxemic respiratory failure, non-invasive respiratory support

## Abstract

COVID-19 patients often present with rapidly progressing acute hypoxemic respiratory failure, requiring orotracheal intubation with different prognostic issues. However, ICU specialists lack predictive tools to stratify these patients. We conducted a single-center cross-sectional retrospective study to evaluate if the ROX index, measured under non-invasive oxygenation support, can predict ICU mortality in a COVID-19 intubated patient cohort. This study took place in the division of intensive care at the Geneva University Hospitals (Geneva, Switzerland). We included all consecutive adult patients treated by non-invasive oxygenation support and requiring intubation for acute respiratory failure due to COVID-19 between 9 September 2020 and 30 March 2021, corresponding to the second local surge of COVID-19 cases. Baseline demographic data, comorbidities, median ROX between H0 and H8, and clinical outcomes were collected. Overall, 82 patients were intubated after failing a non-invasive oxygenation procedure. Women represented 25.6% of the whole cohort. Median age and median BMI were 70 (60–75) years and 28 (25–33), respectively. Before intubation, the median ROX between H0 and H8 was 6.3 (5.0–8.2). In a multivariate analysis, the median ROX H0–H8 was associated with ICU mortality as a protective factor with an odds ratio (95% CI) = 0.77 (0.60–0.99); *p* < 0.05. In intubated COVID-19 patients treated initially by non-invasive oxygenation support for acute respiratory failure, the median ROX H0–H8 could be an interesting predictive factor associated with ICU mortality.

## 1. Introduction

The Coronavirus disease-19 (COVID-19) pandemic is expanding around the globe causing a worldwide healthcare crisis. A common feature of COVID-19 is the progression of hypoxemic respiratory failure leading to acute respiratory distress syndrome [1]. A minority of COVID-19 patients’ (estimated 5%) conditions will become critical in a two-week time frame with a need for intensive care unit (ICU) admission for orotracheal intubation (OTI) and protective lung ventilation [2]. This number represents, however, an important healthcare burden for intensive care units in Europe, where significant disparities exist among countries in terms of ICU resource allocation capacity [3].

The development or upscaling of multidisciplinary intermediate care units (IMCU) has been proposed to relieve ICU units by taking charge of less severe patients and those who may be restricted from mechanical ventilation due to severe concomitant conditions [4,5]. IMCU act as buffers regulating hospital patient flow either as step-up or step-down units [6]. Our institution opted for such a strategy with precise admission criteria at the beginning of the second SARS-CoV2 pandemic wave, allowing the use of high-flow nasal oxygenation (HFNO), continuous positive airway pressure (CPAP), and awake prone positioning under strict continuous monitoring in IMCU, providing time for the majority of severe COVID-19 patients to recover [7,8]. However, this non-invasive strategy might lead to patient self-infected lung injury (P-SILI), a hypothetical pathological process occurring after the application of an excessive mechanical power to residual ventilated lungs under spontaneous ventilation support [9]. This process has been described in experimental and indirect clinical studies, where unregulated ventilatory effort for a prolonged period of time could have a causative role for SARS-CoV2 lung injury progression [10]. 

The ROX index is defined as the ratio of oxygen saturation (SpO2), as measured by pulse oximetry/FIO2, to respiratory rate (RR), and was described by Roca et al. as a predictive factor of mechanical ventilation in patients under HFNO, with a value lower than 4.88 measured 12 h after admission being an indicator that a patient may be at higher risk of orotracheal intubation (OTI) [11]. In a recent systematic review and meta-analysis of eight observational studies, Prakash et al. showed that the ROX index might be an interesting predictor of HFNO failure in COVID-19 patients with acute hypoxemic respiratory failure [12]. The pooled sensitivity and specificity were 0.7 (95% CI, 0.59–0.8) and 0.79 (95% CI, 0.67–0.88), respectively. Cut-off values of ≤5 and >5 had summary areas under the curve of 0.76 (0.72–0.8) and 0.87 (0.83–0.89), respectively. However, 2/8 trials and 1/8 trials were at moderate and high risks of bias, respectively, and the timing of the ROX index measurement ranged from 2 to 12 h, making it unclear what optimal ROX index threshold values should be applied. In addition, doubts remain around whether the ROX index calculated at the time of worsening severe acute hypoxemic respiratory failure might be an early predictor of mortality in later ICU intubated patients. This uncertainty leaves the ICU medical staff in an uncomfortable position with respect to the lack of an important prognostic factor for COVID-19 patients needing mechanical ventilatory support. 

The purpose of this study was to assess whether early ROX index measurement in IMCU in a cohort of severe hypoxemic COVID-19 patients could help predict later ICU mortality after orotracheal intubation. 

## 2. Materials and Methods

This was a retrospective cross-sectional observational study carried out at a university teaching hospital (Hôpitaux Universitaires de Genève, Geneva, Switzerland) from September 2020 to March 2021, a period corresponding to the second local surge of COVID-19 cases. All adult patients with a SARS-CoV2 infection confirmed by oro- or nasopharyngeal reverse transcriptase polymerase chain reaction (RT-PCR) who had been admitted to the ICU and needed orotracheal intubation (OTI) were included. Patients without prior IMCU admission were excluded. The study was approved by the ethics committee of our institution (CCER 2020-00917).

All patients involved in the study were initially admitted into intermediate care units (IMCU) where they received non-invasive respiratory support combining alternatively continuous positive airway pressure (CPAP, Hamilton^®^ T1 ventilators, Bonaduz AG, Bonaduz, Switzerland) and high-flow nasal oxygen support (HFNO, Airvo 2 Optiflow^®^ system, Fisher & Paykel Healthcare, Stafford, TX, USA). This strategy was not protocolized. CPAP tolerance was evaluated by respiratory physiotherapists during CPAP support use. HFNO was initiated with high flows of 50–60 L/min, titrating FiO2 to maintain SpO2 between 90% and 94%. CPAP was delivered with masks, with FiO2 to maintain SpO2 between 90% and 94%, and with a PEEP level of 5–10 cm H20, when tolerated. Awake prone position was left to the discretion of the physician in charge. Non-invasive ventilation was not delivered during patients’ IMCU stay to avoid excessive tidal volumes. 

### 2.1. IMCU and ICU Admission Criteria

A standardized protocol was used as a triage strategy to refer patients to more intensive respiratory units from medical wards [8]. Admission criteria to IMCU were defined as a need of FIO2 > 50% (mostly under venturi mask) to maintain a SpO2 ≥ 90%, without respiratory distress. Admission criteria to the ICU from IMCU (step-up strategy) were defined as SpO2 < 90% despite FiO2 ≥ 80% with HFNO or CPAP and respiratory distress. Invasive airway support was only performed in the ICU and left to the discretion of the physician in charge of the patient.

### 2.2. Data Collection

During patients’ IMCU stay, SpO2 and respiratory rate (RR) were automatically extracted every 15 min from monitors to the electronic patient system (DPI^®^). FiO2 and the time spent under non-invasive respiratory support (HFNO or CPAP) were manually recorded in the latter electronic system by health workers. In order to avoid arbitrary retrospective data selection, and to get as many ROX index values as possible, it was decided to calculate a median ROX index for a convenient period of time from IMCU admission to hour 8. For this purpose, available SpO2, respiratory rate, and FiO2 data with their related time records were extracted for each patient from the electronic patient system to an excel sheet. The ROX index was calculated according to the following formula: ([SpO2/FIO2]/RR) [12]. Only values under HFNO were selected and the median of these values was calculated (Excel^®^) for this period of time. 

Other data extracted from medical records included: demographic data (age, sex), BMI, medical history, APACHEII score, PaO2/FIO2 ratio, mean arterial pressure, heart rate, invasive ventilation length, respiratory system compliance measured after intubation and calculated as the tidal volume (VT) divided by the driving pressure (DP), symptom length until hospitalization, total hospital and ICU length of stay, duration from admission to OTI, and death. 

### 2.3. Outcomes

The primary outcome studied was mortality at ICU discharge. 

### 2.4. Statistical Analysis

The analytical sample comprised all the participants admitted in the ICU from September 2020 to March 2021. Continuous variables were presented as medians and interquartile ranges, and categorical variables were expressed as the number of patients (percentage). We performed descriptive analyses of patients’ characteristics and ROX scores at their admission according to survivor status. To assess the differences between survivors and non-survivors, Chi-square tests were performed for categorical variables and *t*-test for continuous parametric variables. All assumptions were met for normality. To investigate the association between the ROX score and mortality, we performed univariate and multivariate logistic regressions, adjusting the estimates for APACHE2. Two-tailed *p*-values at 0.05 were considered statistically significant. We also performed a multivariate analysis with a stratification of ROX value (<5.5, 5.5–7, >7). All statistical analyses were conducted using STATA version 16.1 (Stata Corp., College Station, TX, USA, 2007). Results are expressed as medians, interquartile ranges (IQR), or odds ratios (OR) with 95% confidence interval (CI 95%).

## 3. Results

**Figure 1:**
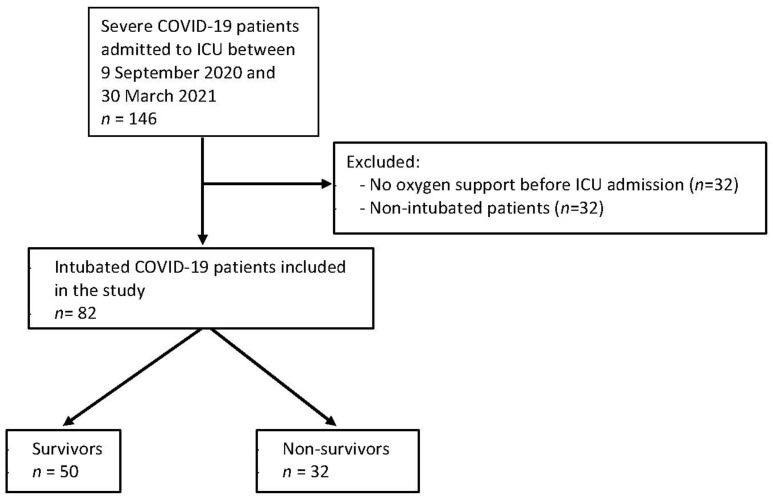
Flow chart study.

A total of 82 patients were included in this study (Figure 1). 

The median (IQR) age of the patients was 70 (60–75) and 26% (21/82) of the patients were female. A history of hypertension was present in 50% (41/82) of the patients, diabetes in 40% (30/82), and hypercholesterolemia in 33% (27/82). The median (IQR) BMI was 28 (25–32). The median number of ROX values available for every patient during the H0–H8 timeframe was 18 (IQR 7; 29). Before ICU admission, the median ROX H0–H8 value under non-invasive procedures was 6.26 [5.04–8.18]. After ICU admission, the median (IQR) duration of invasive mechanical ventilation was 12 (7–21) days, with a median (IQR) ICU length of stay of 13.5 (8–23) days, a median (IQR) IMCU length of stay of 4 (2–7) days, and a median (IQR) total hospital stay of 27 (19–46) days. ICU mortality was 40% (32/82). All baseline characteristics are provided in Table 1. 

### Outcomes

**Figure 2:**
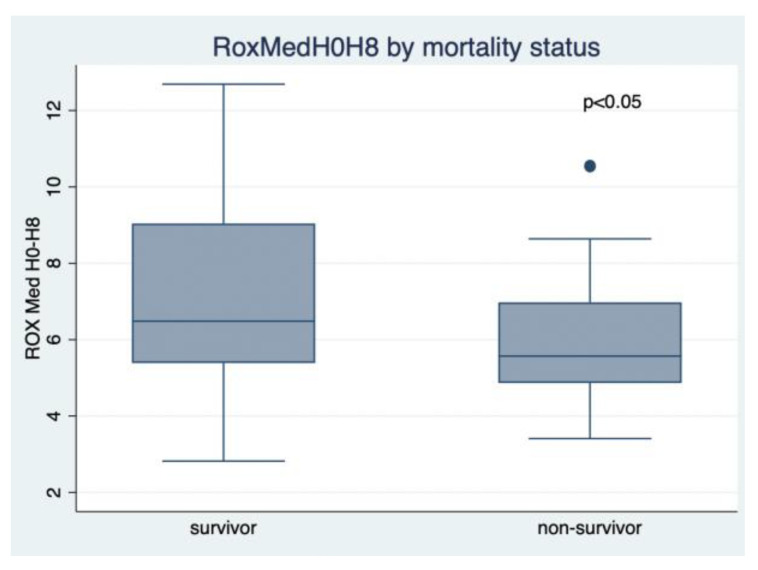
Median Rox index H0H8 by mortality status.

The univariate analysis revealed that surviving patients had a lower median age (68 (59–74) vs. 70.5 (67–76.5); *p* = 0.04), lower APACHE II score (19 (11–28) vs. 27.5 (22.5–30), *p* < 0.01), and higher median ROX H0–H8 score (6.49 (5.39–9.04) vs. 5.57 (4.86–6.97), *p* = 0.04) compared with the non-survivor group, as shown in Table 2 and Figure 2. There was not any difference in the median duration from hospitalization to ICU intubation (3 (2–7) vs. 4.5 (3–8), *p* = 0.23) nor median IMCU length of stay (2.5 (1–4) vs. 2 (1–5), *p* = 0.84) between both groups, as shown in Table 2. 

The multivariate analysis showed the median ROX H0–H8 to be independently associated with ICU mortality as a protective factor, with an odds ratio (OR) (95% CI) = 0.77 (0.60–0.99), *p* < 0.05, and APACHE II (including age) as a risk factor OR (95% CI) = 1.09 (1.02–1.15), *p* < 0.05, as seen in Table 3. 

Finally, a logistic regression model with ROX H0–H8 found a value ≥7 to be the best threshold for predicting survival in our cohort, as shown in Table 4. 

## 4. Discussion

The findings of the present study show that the median ROX values measured during the first 8 h after the initiation of non-invasive strategy could be an interesting independent predictor of ICU mortality in severe COVID-19 patients needing OTI at the end, with 7 points being the best cut-off value for predicting survival. This value results from a comprehensive recording of all the available ROX index measurements in an early medical care time frame. 

The ROX index has been proposed to predict the need for intubation in patients with acute hypoxemic respiratory failure [11]. This index has been assessed in spontaneous-breathing COVID-19 patients, especially as a risk marker of secondary intubation in patients under non-invasive strategies [13,14]. To our knowledge, few studies have assessed the ROX index as a mortality predictive factor. Gianstefani et al. published a prospective study enrolling 554 patients with mild COVID-19 symptoms and found a higher ROX index cut-off value (22.3 point) to be predictive of mortality in an emergency department setting [15]. There was no mention of HFNO use. Other studies evaluating the ROX index found lower threshold values [16,17,18,19,20,21], somehow with a higher mortality in the IOT arm [18,21], with the ROX index being measured later at one or multiple time intervals [16,18,19,20], and with a composite outcome of mortality and mechanical ventilation [19,20]. 

We did not find any association between IMCU length of stay or time from hospital admission to OTI and mortality in this severe respiratory failure patient cohort. Hence, we cannot infer that a faster OTI could improve patient prognosis by limiting the potential deleterious effects of spontaneous breathing as reported in previous studies [10]. This may be due to a lack of power, but was also described in a recent multicenter, retrospective, observational study with similar patient characteristics [16].

In a recent study, 60% of COVID-19 patients with a severe form did not need ICU admission for OTI [3]. This result is mainly associated with the development of IMCU, where non-invasive oxygen support can be used. The development of such structures prevented ICU admission, limiting the shortage of ICU capacity. Indeed, HFNO and CPAP significantly reduced the rate of OTI and ICU hospitalizations in a recent meta-analysis including 3804 patients [22]. Nevertheless, 39% of patients admitted in IMCU needed ICU admission [3]. Delayed intubation has been reported to be associated with a worse outcome [23]. Indirect information suggests that vigorous and dysregulated respiratory effort may be a promoter of lung injury, a phenomenon known as “patient self induced lung injury (P-SILI)” [24,25,26]. Spontaneous breathing can be particularly deleterious [9] in this context. P-SILI could be secondary to two main mechanisms: reduction in functional residual capacity, with the development of atelectasis, and increments in dynamic strain [27]. Several reports have been published about the use of other non-invasive indices of respiratory drive and effort in patients with severe forms of COVID-19 [28] that could potentially help in detecting progressive lung injury. Esophageal pressure change related to inspiratory work of breath is a good proxy of pleural pressure change and represents the gold standard [29]. Maximal transpulmonary pressure measured with an esophageal manometer seems to be independently associated with COVID-19 pneumonia progression [30]. However, esophageal manometers are not easily available and complicated by technical issues, so alternative means have emerged to try to estimate transpulmonary gradients. A small pilot study by Corradi et al. showed that diaphragmatic thickening fraction (DFT) could be a potential predictor of CPAP failure, with the best cut-off DFT value of 21% having a sensitivity of 94% and specificity of 89% [31]. Respiratory variations of the central venous pressure could be used to inform clinicians of potentially large injurious transpulmonary pressure gradients under non-invasive oxygen support that could lead to faster mechanical respiratory control [32]. Central venous pressure swing may perform better than diaphragm ultrasound as a measure of inspiratory effort when evaluated with esophageal pressure in COVID-19 mechanically ventilating patients [33]. The use of mechanical ventilation can prevent or attenuate the damage of the lung setting by better control of volume and frequency under sedation [34]. The measurement of the respiratory drive in patients with COVID-19 ARDS holds potential relevance in the selection of initial ventilator support [35]. The occlusion pressure in the first 100 ms of an occlusion (P0.1) and the maximal deflection in airway pressure from positive end-expiratory pressure during an end-expiratory airway occlusion maneuver have been proposed to identify patients with worsening respiratory function [9]. The latter method has recently been shown to detect high respiratory effort and transpulmonary driving pressures in COVID-19 patients with a moderate degree of correlation with esophageal manometry [36]. These parameters are usually measured under OTI. In spontaneous-breathing patients, the measurement of the drive pressure could be more difficult to realize. In this respect, the ROX index can certainly help us in evaluating non-intubated patient COVID-19 illness severity. 

Our study has several limitations. First, the study focused only on patients who required admission to the ICU for OTI, with worse prognosis by definition, limiting generality. Second, the lack of power in our study yielded large confidence intervals, precluding strong conclusions from our results regarding the clinical effect. Third, oxygen support procedures were not standardized in this retrospective study, and awake prone positioning was delivered according to the physician in charge and was not specifically recorded. Lastly, our study is limited by its cross-sectional design, which does not allow us to infer causality between ROX and mortality because of the potential bias inherent to the non-interventional design. 

## 5. Conclusions

An early ROX index measurement could be an interesting parameter to collect in patients needing OTI to predict ICU mortality and discuss potential ICU care strategies in the scenario of a worldwide ICU bed shortage due to the COVID-19 pandemic. Additional studies are needed to confirm our results and evaluate the performance of a change in strategy (for instance, a faster intubation) according to IMCU ROX evolution in a larger COVID-19 ARDS population. Meanwhile, patients with lower initial median ROX indexes should deserve our full attention at ICU admission in order to improve their mortality outcome. 

## Figures and Tables

**Table 1:** Patients’ characteristics.

Baseline Characteristics	*n*= 82
Sex, women, *n* (%)	21 (25.6%)
Age, median (IQR)	70 (60–75)
BMI, median (IQR)	28.2 (24.7–32.8)
Hypertension, *n* (%)	41 (50%)
Diabetes, *n* (%)	33 (40.2%)
Hypercholesterolemia, *n* (%)	27 (32.9%)
Chronic renal insufficiency, *n* (%)	8 (9.8%)
COPD, *n* (%)	7 (8.5%)
Admission	
ROX Index H0H8, median (IQR)	6.26 (5–8.2)
Duration from admission to OTI in days, median (IQR)	5 (4–7)
IMCU length of stay in days, median (IQR)	2 (1–5)
ICU	
APACHEII, median (IQR)	24 (14–29)
Invasive ventilation in days, median (IQR)	12 (7–21)
Respiratory system compliance after OTI in mL/cm H20, median (IQR)	32.5 (26.9–41.7)
ICU LOS in days, median (IQR)	13.5 (8–23)
Total length of hospital stay in days, median (IQR)	27 (19–46)
Total mortality, *n* (%)	32 (39.9%)

Legend: Values are expressed as numbers and percentages for categorical variables, median values and interquartile ranges (IQR) for continuous variables. BMI—Body Mass Index (kg/m^2^), COPD—chronic obstructive pulmonary disease, ICU—intensive care unit, IMCU—intermediate care unit, LOS—length of stay, OTI—orotracheal intubation, and RoxMedH0H8—Median Rox Index value during H0 to H8 period of time.

**Table 2:** Patients’ characteristics by survivor status.

Baseline Characteristics	Survivors, *n* = 50	Non-Survivors, *n* = 32	*p* Value
Sex, women, *n* (%)	14 (28%)	7 (21.88)	0.54
Age, median (IQR)	68 (59–74)	70.5 (67–76.5)	0.04 *
BMI, median (IQR)	28.95 (25.7–33.5)	27.25 (24.1–29.5)	0.13
Hypertension, *n* (%)	27 (54%)	14 (43.75%)	0.36
Diabetes, *n* (%)	22 (44%)	11 (34.4%)	0.39
Hypercholesterolemia, *n* (%)	16 (32%)	11 (34.38%)	0.82
Chronic renal insufficiency, *n* (%)	4 (8%)	4(8%)	0.50
COPD	3 (6%)	4 (12.5%)	0.30
Admission			
ROX index H0H8, median (IQR)	6.49 (5.39–9.04)	5.57 (4.86–6.97)	0.04 *
Symptoms length until hospitalization in days, median (IQR)	7 (3–8)	5.5 (3–7.5)	0.5
IMCU length of stay in days, median (IQR)	2.5 (1–4)	2 (1–5)	0.84
Duration from admission to OTI in days, median (IQR)	3 (2–7)	4.5 (3–8)	0.23
ICU			
APACHEII, median (IQR)	19 (11–28)	27.5 (22.5–30)	<0.01 *
PaO2/FIO2 ratio in kPa, median (IQR)	12.7 (10.2–16.9)	13 (10.5–14.4)	0.6
Mean Arterial Pressure in mmHg, median (IQR)	91.5 (82–98.7)	85.7 (77.4–96.1)	0.19
Heart Rate in beats/min, median (IQR)	84 (70–96)	91 (76.5–101)	0.43
CRP in mg/L, median (IQR)	135.7 (73.8–219.6)	119.5 (49–216.3)	0.57
Invasive ventilation in days, median (IQR)	10.5 (6–22)	13.5 (8–19.5)	0.54
Respiratory system compliance after OTI in mL/cm H20, median (IQR)	32.3 (24.8–41.7)	34.3 (27.1–44.6)	0.35
ICU LOS in days, median (IQR)	13 (8–23)	15.5 (10–22.5)	0.82
Total hospital stay in days, median (IQR)	32.5 (25–54)	20.5 (14–27)	<0.01 *

Legend: Values are expressed as numbers and percentages; median values and interquartile range (IQR). * *p* < 0.05.

**Table 3:** Univariate and multivariable analyses with ROXmedH0H8, APACHEII score.

	ICU Mortality, OR (95% CI)
RoxMedH0H8	0.78 (0.62–0.99) *
RoxMedH0H8	0.77 (0.6–0.99) *
APACHE2	1.09 (1.02–1.15) *
Sex	0.79 (0.24–2.58)

Legend: Odds ratios and 95% confidence intervals from logistic regression models with the RoxMedH0H8 as predictor, adjusted for APACHEII. * *p* < 0.05

**Table 4:** Multivariable analysis with ROXmedH0H8, APACHE II score.

ROXMedH0H8	ICU Mortality, OR (95% CI)
<5.5	Ref.
5.5–7	0.65 (0.20–2.08)
≥7	0.26 (0.078–0.89) *
APACHEII	1.09 (1.02–1.16) *
Sex	0.79 (0.24–2.58)

Legend: Odds ratios and 95% confidence intervals from logistic regression models with the RoxMedH0H8 as predictor, adjusted for APACHEII. * *p* < 0.05

## Data Availability

The datasets used and analyzed during the current study are available from the corresponding author on reasonable request.
